# Mutant Amyloid Precursor Protein Differentially Alters Adipose Biology under Obesogenic and Non-Obesogenic Conditions

**DOI:** 10.1371/journal.pone.0043193

**Published:** 2012-08-17

**Authors:** Linnea R. Freeman, Le Zhang, Kalavathi Dasuri, Sun-Ok Fernandez-Kim, Annadora J. Bruce-Keller, Jeffrey N. Keller

**Affiliations:** Pennington Biomedical Research Center/LSU System, Baton Rouge, Louisiana, United States of America; Oregon Health & Science University, United States of America

## Abstract

Mutations in amyloid precursor protein (APP) have been most intensely studied in brain tissue for their link to Alzheimer’s disease (AD) pathology. However, APP is highly expressed in a variety of tissues including adipose tissue, where APP is also known to exhibit increased expression in response to obesity. In our current study, we analyzed the effects of mutant APP (E693Q, D694N, K670N/M671L) expression toward multiple aspects of adipose tissue homeostasis. These data reveal significant hypoleptinemia, decreased adiposity, and reduced adipocyte size in response to mutant APP, and this was fully reversed upon high fat diet administration. Additionally, mutant APP was observed to significantly exacerbate insulin resistance, triglyceride elevations, and macrophage infiltration of adipose tissue in response to a high fat diet. Taken together, these data have significant implications for linking mutant APP expression to adipose tissue dysfunction and global changes in endocrine and metabolic function under both obesogenic and non-obesogenic conditions.

## Introduction

Amyloid precursor protein (APP) is expressed in a variety of tissues including brain, skeletal muscle, adipose tissue, and testes [1]–[5]. Most studies for APP have focused on its link to the pathogenesis of Alzheimer’s disease (AD), with the cleavage products of APP known to be a significant component of amyloid plaques observed in both the aging and AD brain [6]–[8]. Similarly, APP is involved in the generation of proteinaceous inclusions observed in muscle tissue as the result of inclusion body myositis [9], [10]. The biological role for APP in adipose tissue has not yet been well established, although APP is known to be increased in adipose cells in response to obesity in humans and mice [1], [2], [11]. The specific contributions of APP to the complications of obesity remain largely undefined. Furthermore, the impact of APP mutations associated with AD and cerebral amyloid angiopathy (CAA) [12]–[14] have not been well established in adipose tissue. Understanding the potential for APP, and mutant APP expression, to modulate specific aspects of adipose-associated endocrine and metabolic function may provide novel insight as to how peripheral APP expression contributes to the global physiological changes, and potentially the modulation of brain homeostasis.

It was once thought that fat was largely an inert tissue, with adipose tissue only peripherally linked to metabolism. We now know that adipose tissue is essential to the regulation of energy homeostasis under physiological conditions (balancing energy homeostasis in response to energy expenditure and energy intake) [11], [15], and contributes to metabolic disease (insulin resistance) in response to obesity [16], [17]. In both of these paradigms, adipose tissue mediates its effects on the body via the secretion of adipokines [11], [18], [19], and through sequestration and release of energy substrates including fatty acids and glucose [11], [20].

In the current study we analyzed multiple aspects of adipose biology, adipokine signaling, and insulin resistance using an established mouse model of CAA [21]–[23]. This mouse model utilizes the Thy-1 promoter to drive mutant APP (E693Q, D694N, K670N/M671L) expression which is abundantly expressed in brain and adipose tissue [21], [24], [25]. Under non-obesogenic dietary conditions, the expression of mutant APP in adipose tissue resulted in severe hypoleptinemia, significantly decreased adipocyte size, and an overall decrease in the amount of adipose tissue. Interestingly, under obesogenic conditions there was a complete reversal of each of these mutant APP-associated effects, and a concomitant exacerbation of obesity-induced insulin resistance, triglyceride elevation, and macrophage infiltration. This study identifies novel effects of mutant APP expression towards adipose tissue under obesogenic and non-obesogenic conditions, and provides a new model whereby peripheral expression of mutant APP could contribute to brain pathogenesis and the complications of obesity.

## Materials and Methods

### Animals and Dietary Treatments

All animal experiments were approved by the Institutional Animal Care and Use Committee of Pennington Biomedical Research Center. Male and female C57Bl/6 mice (“Control”; Charles River Laboratories) and male and female Tg-SwDI (“CAA”) mice were studied from 2–3 months old until 12–14 months old. The TgSwDI mouse was generated as described previously by Van Nostrand et al. [21]. The CAA and corresponding control mice were maintained and genotyped as described previously [21]–[23], [26]. Briefly, the transgenic mouse model expressed the human AβPP770 isoform including the Swedish, Dutch and Iowa mutations under the Thy-1 promoter. Both genotypes were randomly divided and assigned to either a high fat diet (HF; D12492, Research Diets) or control diet (CD; D12450B) at 2–3 months of age. The HF diet provided 60% kcals from fat while the CD provided 10% kcals from fat. The CD was developed as an appropriate, balanced control to the HF diet: both contained the necessary mineral and vitamin mixes, 20% kcals from protein and the fat source was soybean oil and lard. These diets were administered for 9–11 months. Mice were housed in standard caging with a 12∶12 light/dark cycle and ad libitum access to water and their respective diet unless otherwise noted. Total body fat and muscle content was measured via nuclear magnetic resonance (NMR) spectroscopy (Minispec, Brucker Optics, Billerica MA) and a glucose tolerance test (GTT) was performed before conclusion of the experiment.

**Figure 1:**
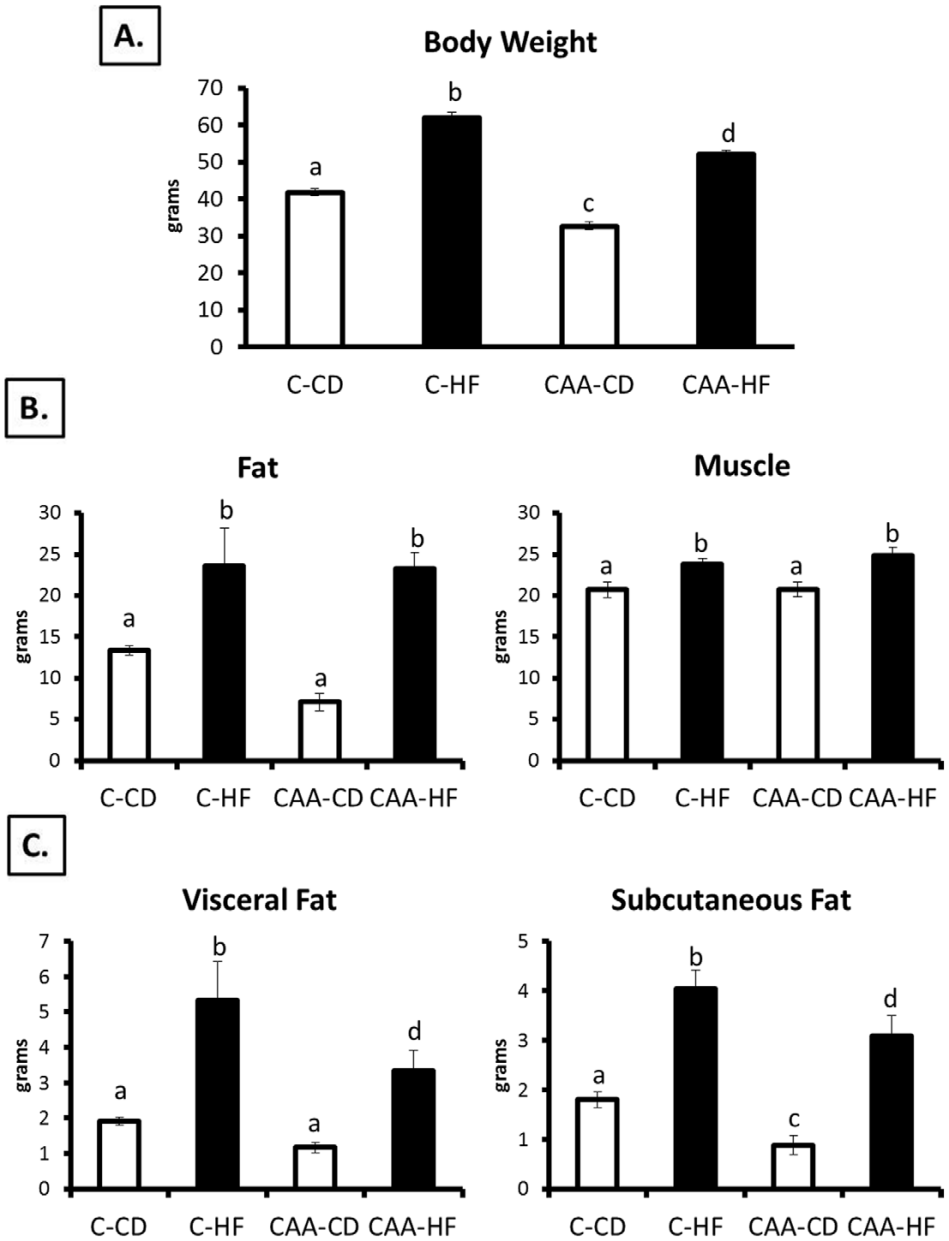
Body weights and body composition. C-HF mice were significantly heavier than all other groups (p<0.0001) while CAA-CD mice weighed significantly less than all other groups (p<0.0001; Figure 1A). Body composition as measured by NMR revealed increased total body fat and muscle (p<0.05) for both groups fed the HF diet (Figure 1B). The CAA-CD mice had less fat compared to all other groups but comparable muscle content to C-CD mice. C-HF had significantly greater visceral and subcutaneous fat pad weights compared to all groups. CAA-CD had less visceral fat and significantly less subcutaneous fat compared to all other groups (p<0.05; Figure 1C).

### Serum and Tissue Collection

At 12–14 months of age, mice were fasted overnight, euthanized by isoflurane anesthesia, exsanguinated via cardiac puncture, perfused with phosphate buffered saline (PBS, pH 8.0) and decapitated. Visceral fat (epididymal fat pads) and subcutaneous fat (inguinal fat pads) were collected, weighed, and then divided between formalin fixation and freezing for biochemical measures.

**Figure 2:**
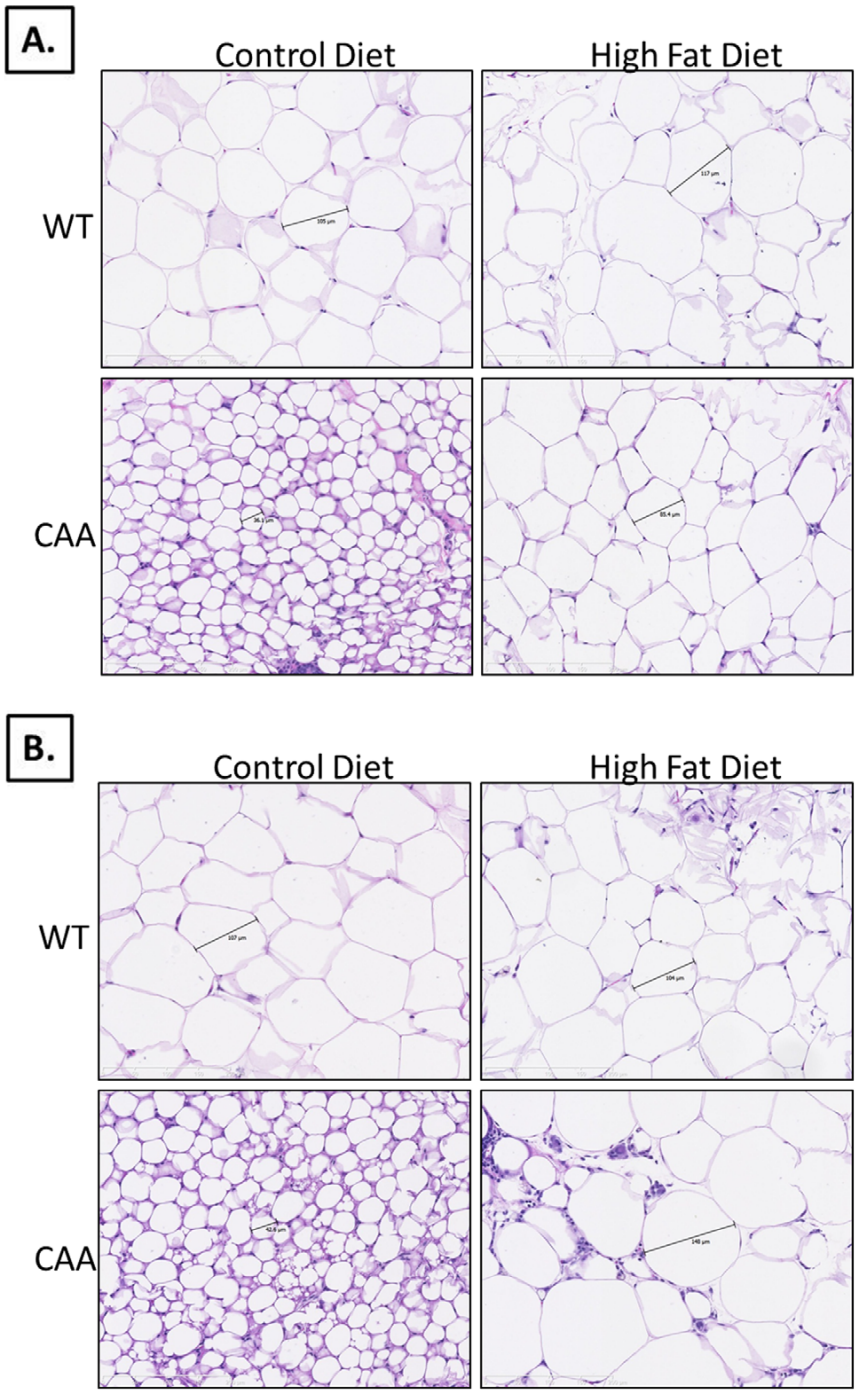
Histology of adipose depots. CAA-CD mice revealed significantly smaller adipocytes compared to all other groups for both subcutaneous (Figure 2A) and visceral (Figure 2B) fat depots. CAA-HF mice had increased adipocyte size compared to their control-fed counterparts, which were more comparable to Control mice, but revealed some signs of inflammation and fibrosis, especially in the visceral fat.

### Histology

The visceral and subcutaneous fat samples were kept in formalin for 10–12 days and then processed for paraffin embedding. Samples were sectioned at 5 µm and then stained with Hemotoxylin & Eosin. Slides were scanned using a Hamamatsu NanoZoomer Digital Slide Scanning System (Hamamatsu City, Japan) at 20X magnification.

**Figure 3:**
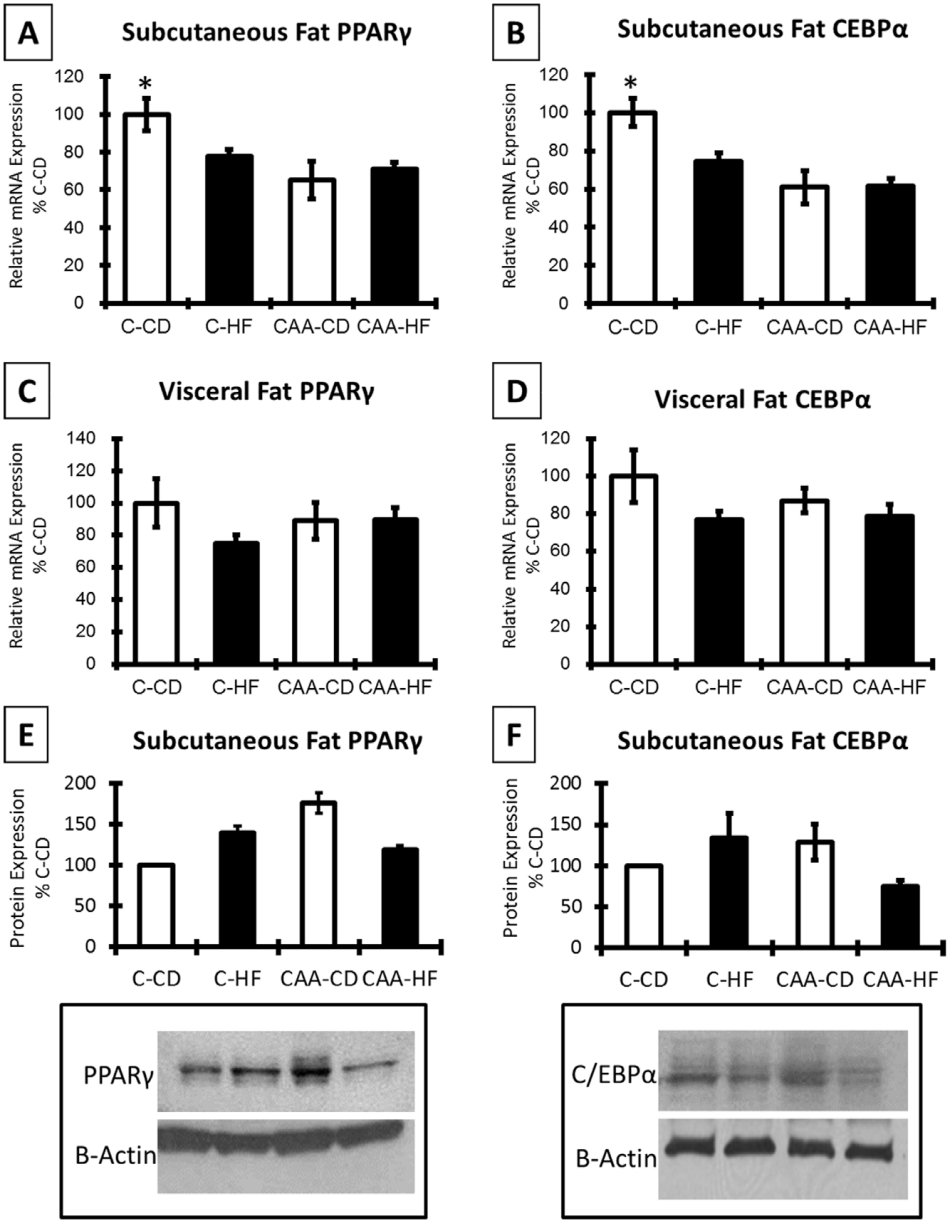
PPARγ and C/EBPα in fat depots. PPAR γ and C/EBPα mRNA levels were significantly higher in C-CD subcutaneous fat compared to all other groups (p<0.05; Figure 3A and B, respectively). However, no significant differences in visceral fat were determined (Figure 3C and D) or western blot analysis of subcutaneous fat samples (Figure 3E and F).

### Immunohistochemistry

Visceral and subcutaneous fat samples were also analyzed using immunohistochemistry. Paraffin was removed by washing slides three times in xylenes, tissue was re-hydrated in a series of alcohol washes (100%, 95%, 70%, and 50%), and then tissue was treated for heat-mediated antigen retrieval. Slides were washed, blocked in serum and incubated overnight with primary antibody (Iba-1 antibody for macrophages (1∶300, Wako, Osaka, Japan)). The next day slides were washed, incubated with secondary antibody (Vector, Burlingame, CA), and developed using VIP (Vector, Burlingame, CA). Slides were scanned using a Hamamatsu NanoZoomer Digital Slide Scanning System (Hamamatsu City, Japan) at 20X magnification.

**Figure 4:**
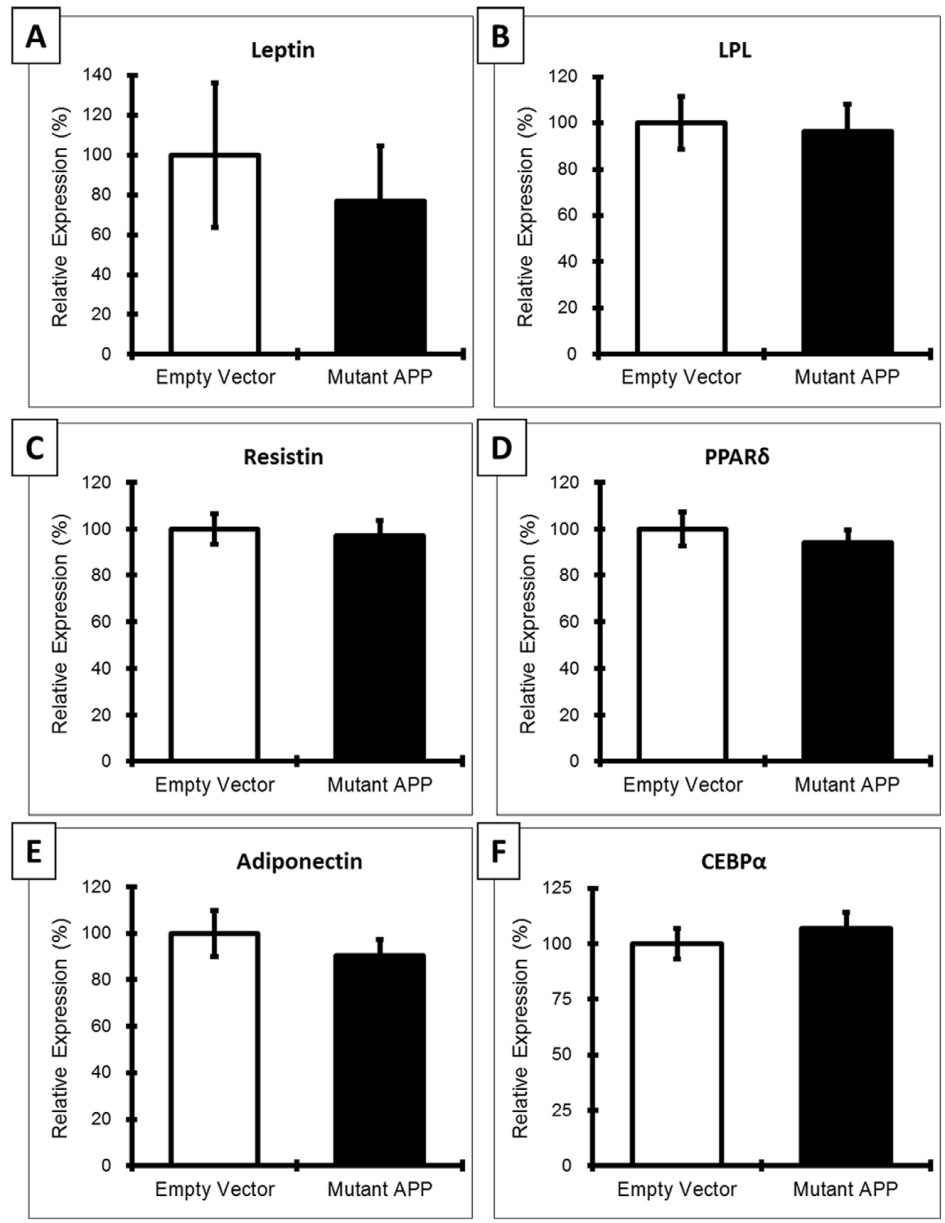
Analysis of 3T3-L1 preadipocytes expressing mutant APP. No significant differences in adipokines, differentiation factors, or lipases (Figure 4A–F) were determined between preadipocytes expressing mutant APP and empty vector. However, reduced leptin was observed for adipocytes expressing mutant APP (Figure 4A).

### Serum Analysis

Blood collected at euthanization via cardiac puncture was allowed to clot overnight and then centrifuged. Serum was isolated and analyzed using ELISA for: Leptin (R&D Systems, Minneapolis, MN), Resistin (R&D Systems, Minneapolis, MN), and Adiponectin (R&D Systems, Minneapolis, MN). Briefly, capture antibody was incubated on the 96-well microplate overnight, serum samples and standards were loaded in duplicate the next day, detection antibody was applied and incubated for 2 hours and then Streptavidin-horseradish peroxidase (HRP) and Tetramethyl Benzidine (TMB; Life Technologies, Grand Island, NY) chromogen were used to catalyze the color change reaction. Plates were read at 450 nm with wavelength correction set to 570 nm. Serum was also analyzed using a quantitative colorimetric kit for triglycerides measurement (Wako, Osaka, Japan).

**Figure 5:**
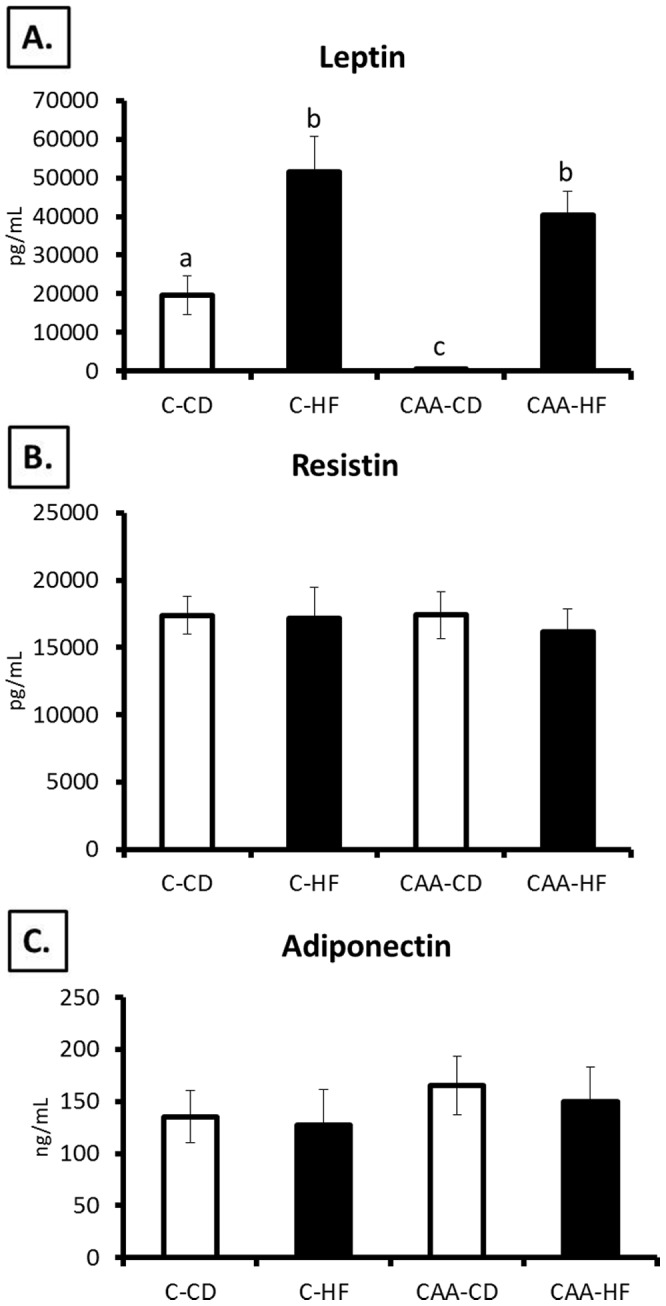
Serum adipokines. CAA-CD mice revealed significant hypoleptinemia (C-CD to CAA-CD: p = 0.019; C-HF to CAA-CD: p<0.0001; CAA-HF to CAA-CD: p<0.0001; Figure 5A). This was reversed by HF diet treatment as C-HF and CAA-HF had comparable leptin levels, which were also significantly higher than C-CD mice (p<0.0001 and p = 0.015, respectively). No significant differences for the adipokines resistin (Figure 3B) and adiponectin (Figure 3C) were observed between groups using ELISA serum analysis.

### Cell Cloning and Transfection

Genomic DNA from CAA mice was extracted from the tails using QIAprep® Spin Miniprep Kit (Qiagen, Cat. No. 27104) according to the manufacturer’s instructions. Mutated human APP770 (hAPP770) was PCR amplified using Genomic DNA from the CAA mouse as a DNA template and using Phusion® High-Fidelity DNA Polymerase (NEB, Cat. No. M0530) for PCR amplification, with primer pairs: ATAAGAATGCGGCCGCATGCTGCCCGGTTTGGCAC (forward) and ACGCGTCGACGGTCTAGTTCTGCATCTGCTCAAAGAACTTGTAGG (reversal). The PCR product was then treated by *Not*I and *Sal*I and inserted into the *Not*I-*Sal*I site of the pCMV-Script vector (Agilent Technologies, Inc. Cat. No. 212220). Both empty vector and vector-hAPP770 were amplified in DH-5α cells (Strategene). The integrity of the hAPP770 DNA insert and the presence of Swedish/Dutch/Iowa mutations were confirmed by nucleotide sequencing. Transfection of empty vector and vector-hAPP770 into 3T3-L1 preadipocytes was performed as described previously [27]. Briefly, 10 µg of DNA and 25 µL of Lipofectamine™ 2000 transfection reagents were diluted into MEM medium at a final volume of 1.25 mL for each and mixed within 5 minutes. The resulting transfection reagent-DNA mixture was allowed to further complex for 15–20 minutes at 22°C and then added into 10 mLs of 3T3-L1 preadipocyte suspension in 3T3-L1 preadipocyte growth medium (DMEM high glucose medium, 10% calf serum, 100 units/ml penicillin G and 100 µg/ml streptomycin), obtained from a 75 cm^2^-flask of 3T3-L1 preadipocyte of 80% confluency in the fast-growth period. The cell mix was then distributed into a 12-well plate at 1 mL/well. 24 hours following transfection, the medium of 3T3-L1 preadipocytes was replaced by 3T3-L1 preadipocyte growth medium for future induction of differentiation.

**Figure 6:**
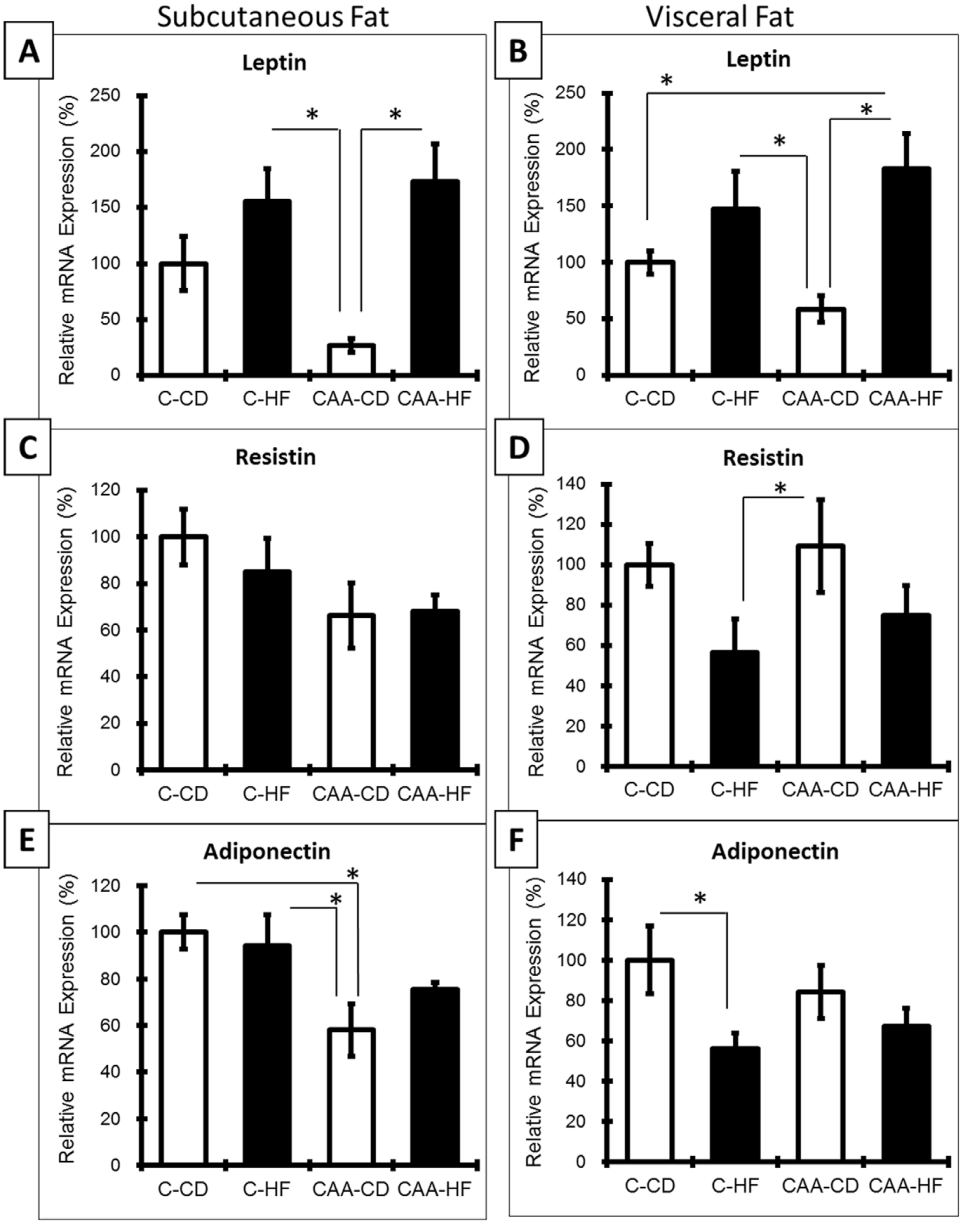
Adipokine mRNA in adipose depots. Both fat depots revealed an increase in leptin for mice fed the HF diet (Figure 6A and B). A significant increase in leptin was determined for CAA-HF mice compared to C-CD and CAA-CD in visceral fat. CAA-CD mice had less leptin expression in both fat pads compared to all other groups. C-HF mice had significantly less resistin expression in visceral fat compared to CAA-CD (p = 0.039; Figure 6D), CAA-CD mice had significantly less adiponectin expression compared to C-CD and C-HF mice in subcutaneous fat (p = 0.005 and p = 0.014, respectively; Figure 6E), and C-HF mice had significantly less adiponectin expression in visceral fat compared to C-CD (p = 0.020; Figure 6F).

### Cell Culture

Murine 3T3-L1 preadipocytes were cultured in the 3T3-L1 preadipocyte growth medium. The medium was changed every 48 hours. To obtain fully differentiated adipocytes, the 3T3-L1 preadipocytes were grown to 2 days post-confluence and induced to differentiate by changing the medium to DMEM high glucose medium containing 10% FBS and 0.5 mM IBMX, 1 µM dexamethasone, 1.7 µM insulin (MDI) and antibiotics (100 units/ml penicillin G and 100 µg/ml streptomycin). After 48 hours this medium was replaced with DMEM high glucose medium supplemented with 10% FBS, penicillin/strepomycin and 0.425 µM insulin. The media was replaced every 2 days thereafter using DMEM high glucose, 10% FBS medium and antibiotics. Cells were fully differentiated by 6 days; they were collected 7–10 days post-MDI treatment for further analysis.

**Figure 7:**
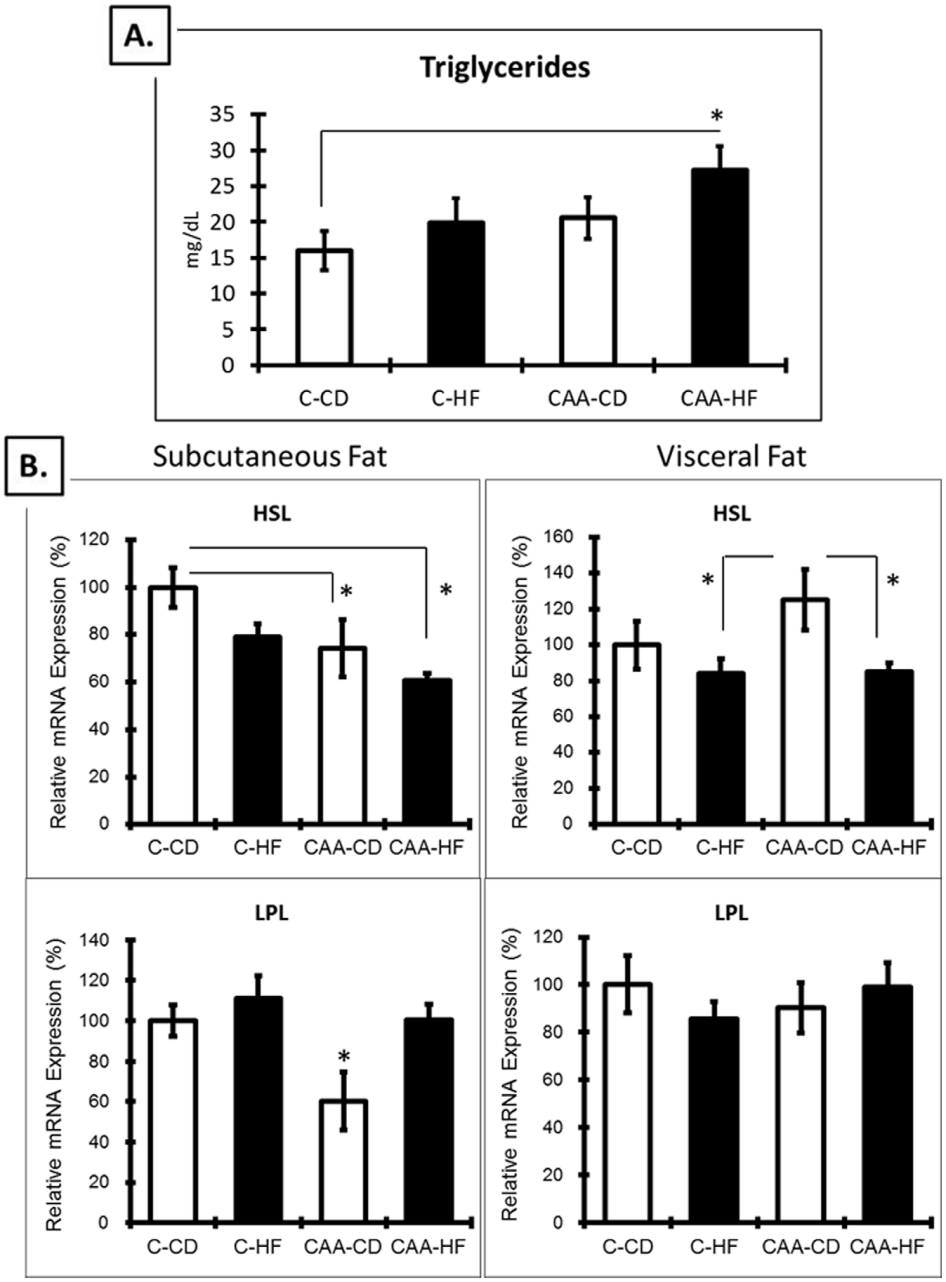
Serum triglycerides and lipase mRNA in adipose. CAA-HF-fed mice had significantly higher triglyceride levels compared to C-CD mice (p = 0.011, Figure 7A). A significant downregulation of HSL mRNA was found in subcutaneous fat for the CAA-CD and CAA-HF compared to the C-CD (CAA-CD to C-CD: p = 0.033, CAA-HF to C-CD: p = 0.002; Figure 7B). On the other hand, CAA-CD mice exhibited increased HSL in visceral fat compared to the C-HF and CAA-HF mice (p = 0.023 and p = 0.026, respectively). The CAA-CD mice had significantly less LPL mRNA expression in the subcutaneous fat compared to all other groups, but no significant differences for LPL were found in the visceral fat.

### RT-PCR

RT-PCR experiments were performed as done previously by our laboratory [27]. Total RNA from 3T3-L1 adipocytes was isolated using RNeasy Mini Kit (Qiagen, Valencia, CA) following the manufacturer’s instructions with minor modifications. After cell harvest and homogenization through a blunt 20-gauge needle in the RLT lysis buffer, the lysate was centrifuged at 15,000×*g* at 4°C for 15 minutes. The liquid layer beneath the frozen fat layer was carefully extracted and used for total RNA extraction. Total RNA from mouse subcutaneous and visceral fat tissue was isolated using RNeasy Lipid Tissue Mini Kit (Qiagen, Valencia, CA) according to the manufacturer’s instructions. The corresponding cDNA was made from 2 µg of extracted total RNA by M-MuLV transcriptase (New England Biolabs, Ipswich, MA) using 20 µL of the reverse transcription system according to the manufacturer’s instructions. For quantitative real-time PCR (qPCR) analysis, aliquots of cDNA were subjected to qPCR in 20 µl of 1×Brilliant II QPCR & QRT-PCR Reagents (Agilent Technologies, Santa Clara, CA), 1× primers and TaqMan probe (6-FAM/ZEN/IBFQ mode) and 10 ng of cDNA. Primers and probes for HSL, LPL, leptin, adiponectin, resistin, PPAR-γ, and CEBP-α were ordered from Integrated DNA Technologies (IDT, Coralville, IA) and PrimeTime Pre-designed qPCR Assays system. Each sample was loaded in triplicate, negative and positive controls were included. Amplification of GADPH was used as an internal reference gene. PCR amplifications were performed as follows: 50°C for 2 minutes, 95°C for 10 minutes, and 40 cycles each with 95°C for 15 seconds and 60°C for 45 seconds using an ABI PRISM 7000 sequence detector according to the manufacturer’s instructions (Applied Biosystems, Foster City, CA). For data analysis, the _ΔΔ_C_t_ method was used. Relative mRNA expression of each gene was expressed as the mean and S.E.M. of 6 independent total RNA extraction and real-time PCR analyses.

**Figure 8:**
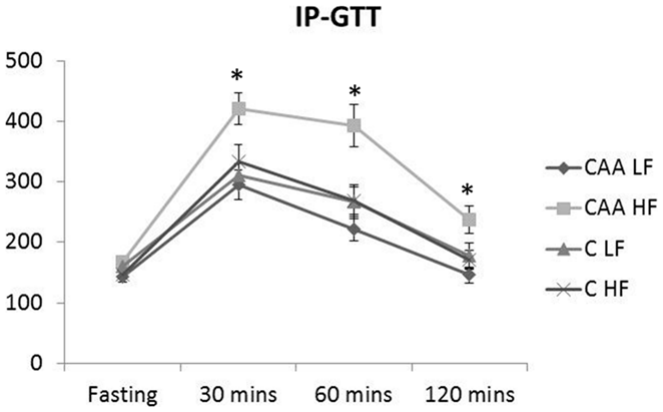
IP-GTT. The CAA-HF-fed mice exhibited a significant increase in glucose levels throughout the test compared to all other groups (p<0.01; Figure 8).

### Western Blotting

Protein lysates from subcutaneous fat samples were made in RIPA buffer using a tissue homogenizer and the amount of protein in lysates was estimated using BCA reagent (Thermo Fisher Scientific, Rockford, IL, USA). Protein samples were analyzed by SDS-PAGE and then transferred onto a nitrocellulose membrane. The membrane was then probed with antibodies as specified by the manufacturer. Antibodies against C/EBPα and PPARγ were purchased from Cell Signaling Technology, Incorporated (Danvers, MA, USA). All electrophoresis and immunoblot reagents were purchased from Bio-Rad Laboratories (Hercules, CA, USA). The HRP-conjugated secondary antibodies were purchased from Vector Laboratories, USA.

**Figure 9:**
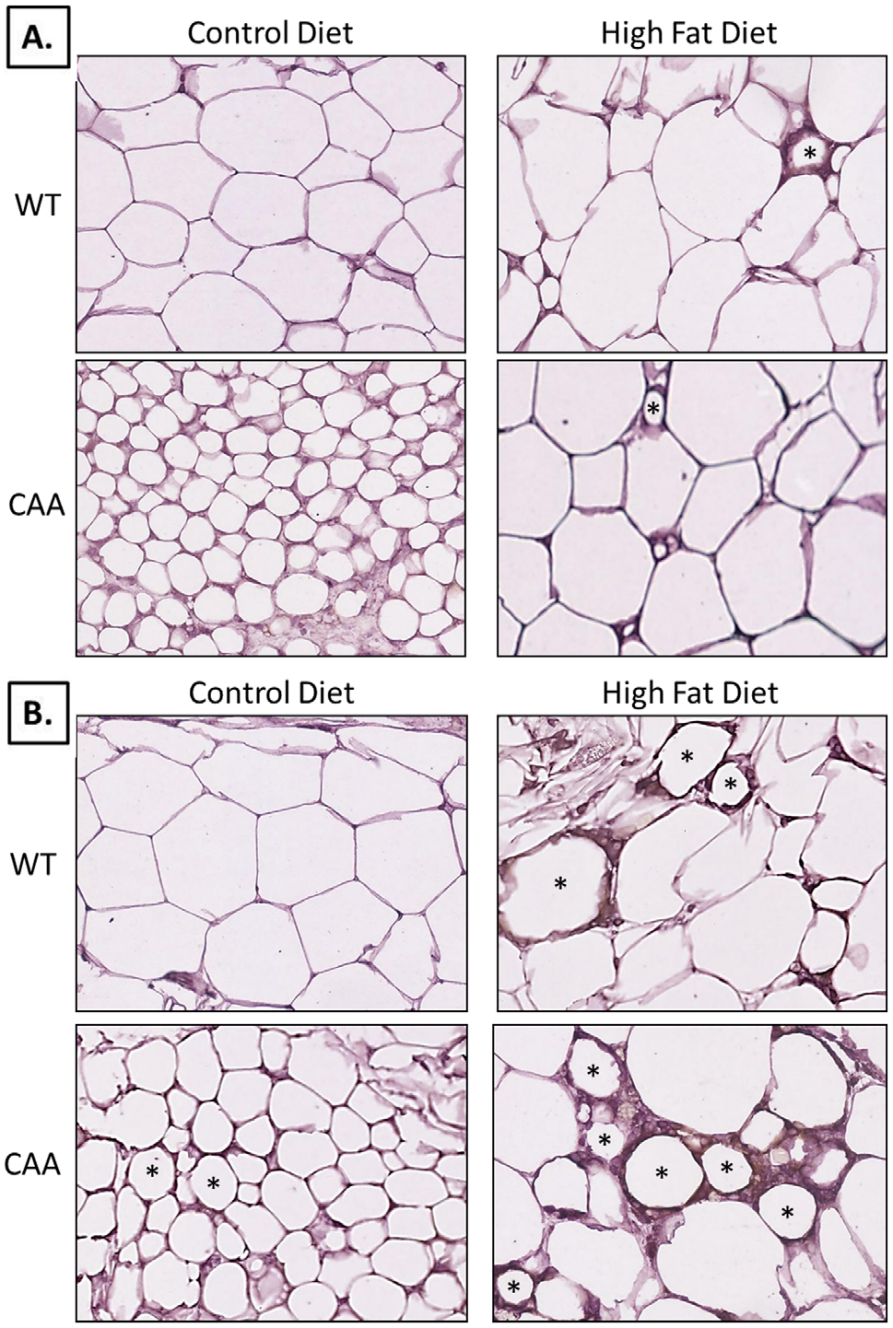
Macrophage infiltration of adipose depots. In subcutaneous fat, the C-HF and CAA-HF revealed the most “crown-like structures”. These were rarely found in the C-CD or CAA-CD subcutaneous fat samples (Figure 9A). Visceral fat depots revealed a greater number of macrophages and crown –like structures (Figure 9B). They were found in CAA-CD, C-HF, and CAA-HF, and rarely found in the C-CD samples. Asterisks mark cells with crown-like structures.

### Statistical Analysis

All data are shown as mean ± standard error of measurement. All 2×2 measures were analyzed by 2-way ANOVA with Fisher’s LSD post hoc analysis (SPSS©) in order to determine differences between HF diet and CD for both genotypes, while cell culture studies were analyzed using unpaired t-tests. Statistical significance for all analyses was accepted at p<0.05 (labeled as *), and labels including “a, b, c, d” refer to statistical significance between the respective groups labeled.

**Figure 10:**
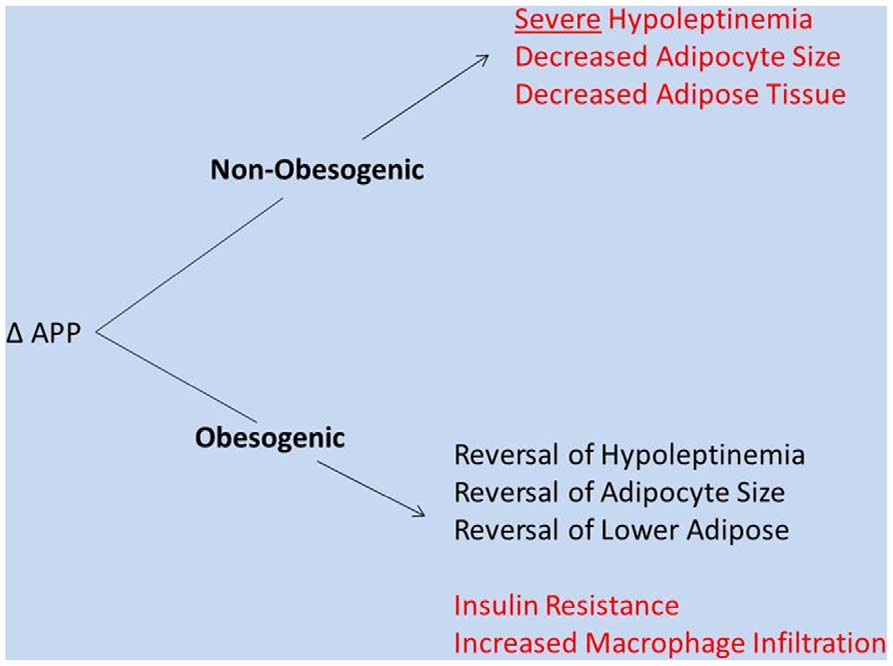
Overview of mutant APP effects on adipose biology. The effects of mutant APP on adipose tissue include: severe hypoleptinemia, decreased adiposity, and reduced adipocyte size. These effects were reversed under obesogenic conditions, but this reversal was accompanied by mutant APP-associated increases in insulin resistance, increased circulating triglycerides, and increased macrophage infiltration of visceral adipose tissue.

## Results

### Body Weights and Body Composition

We determined the differences in total body weights between Control and CAA mice fed the CD or HF diet (Figure 1A). The main effects of genotype and diet were significant (F(1,152) = 68.281; p<0.0001 and F(1,152) = 287.336; p<0.0001, respectively).There was not a significant interaction of Genotype × Diet. Control HF (C-HF) mice were significantly heavier than all other groups (p<0.0001) while CAA-CD mice weighed significantly less than all other groups (p<0.0001). The CAA-HF mice gained weight and were heavier than CAA-CD, but they did not gain as much weight as C-HF mice fed the same diet for the same length of time. Body composition as measured by NMR revealed significant changes to fat and muscle composition due to diet (main effect for diet: F(1,44) = 34.691; p<0.0001 and F(1,44) = 16.530; p<0.0001, respectively). The CAA-CD mice had significantly less fat compared to both HF-fed groups (p<0.0001) and less (but not significant (p = 0.055)) fat compared to Control CD (C-CD) mice. The CAA-CD mice had comparable muscle content to C-CD mice (Figure 1B). At euthanization, fat pads were removed and weighed in order to determine a more specific measure of fat content. A two-way ANOVA revealed significant changes to subcutaneous (main effect of genotype: F(1,52) = 5.684; p = 0.021 and main effect of diet: F(1,52) = 23.599; p<0.0001) and visceral fat pads (main effect of genotype: F(1,52) = 10.508; p = 0.002 and main effect of diet: F(1,52) = 59.273; p<0.0001). C-HF and CAA-HF had similar visceral and subcutaneous fat pad weights. CAA-CD had significantly less visceral and subcutaneous fat compared to the high fat-fed mice (p<0.0001; Figure 1C) and less subcutaneous fat compared to C-CD mice.

### Histological Examination of Adipose

After determining differences in total body weight and fat composition between each experimental group, subcutaneous (Figure 2A) and visceral fat (Figure 2B) samples were analyzed histologically for adipocyte size using Hematoxylin & Eosin staining. Most notable was the drastically smaller adipocyte size for CAA-CD mice in subcutaneous and visceral fat. In subcutaneous fat, C-CD adipocyte size was 100.2±6.3 µm whereas CAA-CD adipocyte size was 51.9±5.2 µm. In response to HF diet treatment, control mice had an average adipocyte size of 93.9±4.3 µm and CAA mice had an average of 107.8±5.1 µm. In visceral fat, C-CD adipocyte size was 107.6±6.4 whereas CAA-CD adipocyte size was 60.2±5.2 µm. In response to HF diet treatment, control mice had an average adipocyte size of 109.9±6.7 µm and CAA mice had an average of 113.6±10.7 µm.

### Analysis of PPARγ and C/EBPα in Fat Depots

In order to further measure the effects of mutant APP on proliferation and/or differentiation of adipocytes, we measured PPARγ and C/EBPα in visceral and subcutaneous fat depots (Figure 3). Subcutaneous fat mRNA levels of these proteins revealed significant differences between groups (main effect of genotype: PPARγ: F(1,20) = 8.757; p = 0.008 and C/EBPα: F(1,20) = 15.934; p = 0.001). Post hoc analysis revealed significantly greater expression of both proteins for C-CD mice compared to all other groups (p<0.05; Figure 3A and B). However, no significant differences in PPARγ or C/EBPα expression were observed in visceral fat (Figure 3C and D) or western blot analysis of subcutaneous fat samples (Figure 3E and F).

### Analysis of Adipokines and Differentiation Factors in 3T3-L1 Preadipocytes

3T3-L1preadipocytes were also analyzed for changes to adipokines and differentiation factors in response to the expression of mutant APP (Figure 4). These analyses revealed no statistically significant differences in gene expression in response to mutant APP; however less leptin (Figure 4A) was measured in preadipocytes containing the mutant APP vector as compared to those with empty vector.

### Circulating Adipokine Levels

In order to determine whether CAA mice exhibited altered lipid and adipokine levels, we analyzed the serum from fasting CAA and control mice (Figure 5). Significant differences in serum leptin between groups were observed due to genotype (F(1,33) = 6.783; p = 0.014) and diet (F(1,33) = 37.568; p<0.0001). Most notable was the severe hypoleptinemia observed in CAA-CD mice (C-CD to CAA-CD: p = 0.019; C-HF to CAA-CD: p<0.0001; CAA-HF to CAA-CD: p<0.0001; Figure 5A). Interestingly, hypoleptinemia in CAA mice was reversed by HF diet treatment, as C-HF and CAA-HF had comparable leptin levels, which were also significantly higher than C-CD mice (p<0.0001 and p = 0.015, respectively). On the other hand, no significant differences for the adipokines resistin (Figure 5B) and adiponectin (Figure 5C) were observed between groups using ELISA serum analysis. CAA-HF-fed mice had significantly higher triglyceride levels compared to C-CD mice (p = 0.011).

### Analysis of Adipokine Expression in Adipose Tissue

To complement serum analysis of adipokines, fat tissue was also analyzed via RT-PCR for leptin, resistin and adiponectin expression (n = 6/group; Figure 6). In agreement with serum analysis results, both subcutaneous and visceral depots revealed significant changes to leptin expression due to diet (main effect for diet in subcutaneous fat: F(1,20) = 15.605; p = 0.001 and visceral fat: F(1,20) = 12.685; p = 0.002) (Figure 6A and B). Additionally, CAA-CD had less leptin in subcutaneous and visceral fat compared to C-CD mice, and a significant increase in leptin was determined for CAA-HF mice compared to C-CD in visceral fat (p = 0.025). While serum analysis of resistin and adiponectin did not reveal any significant differences between groups, mRNA expression was significantly different for these measures: CAA-CD mice had significantly less adiponectin expression compared to C-CD and C-HF mice in subcutaneous fat (p = 0.005 and p = 0.014, respectively; Figure 6E), and C-HF mice had significantly less adiponectin expression in visceral fat compared to C-CD (p = 0.020; Figure 6F). No significant differences in subcutaneous resistin mRNA levels were determined, but CAA-CD had significantly higher levels compared to C-HF in visceral fat (p = 0.039).

### Analysis of Lipase Expression in Adipose Tissue

Further analysis of subcutaneous fat and visceral fat via RT-PCR was performed in order to measure hormone sensitive lipase (HSL) and lipoprotein lipase (LPL) (Figure 7B; n = 6/group). Here, we found significant changes to HSL mRNA expression in subcutaneous fat due to genotype (F(1,20) = 7.706; p = 0.012) and diet (F(1,20) = 4.723; p = 0.042), significant changes to LPL mRNA expression in visceral fat due to genotype (F(1,20) = 5.534; p = 0.029) and diet (F(1,20) = 5.665; p = 0.027), and significant changes to HSL mRNA expression in visceral fat due to diet (F(1,20) = 5.624; p = 0.028). The CAA-CD and CAA-HF mice had significantly less HSL mRNA expression compared to the C-CD mice (CAA-CD to C-CD: p = 0.033, CAA-HF to C-CD: p = 0.002). On the other hand, CAA-CD mice exhibited increased HSL in visceral fat compared to the C-HF (p = 0.023) and CAA-HF mice (p = 0.026). The CAA-CD mice had significantly less LPL mRNA expression in the subcutaneous fat compared to all other groups (p<0.05), but no significant differences for LPL were found in the visceral fat.

### Analysis of Insulin Resistance

Insulin resistance was measured via glucose tolerance test using intraperitoneal (i.p.) injection of glucose (IP-GTT, Figure 8). A significant change in glucose levels was determined due to diet (F(1,44) = 7.448; p = 0.009) with a significant genotype × diet interaction (F(1,44) = 7.028; p = 0.011). The CAA-HF-fed mice exhibited a significant increase in glucose levels throughout the test compared to all other groups (p<0.01). These studies demonstrate mutant APP expression exacerbating HF-induced insulin resistance.

### Increased Macrophage Infiltration in Adipose Tissue

Lastly, we used immunohistochemistry to visualize macrophages in subcutaneous (Figure 9A) and visceral (Figure 9B) adipose tissue. In subcutaneous fat, the C-HF and CAA-HF revealed “crown-like structures” which are adipocytes surrounded by a great number of macrophages, revealing a dead or dying cell. These were rarely found in the C-CD or CAA-CD subcutaneous fat samples. However, it is important to note the presence of fibrotic tissue in CAA-CD mice which was invaded by macrophages (as seen at the bottom of the CAA-CD representative image). Visceral fat depots revealed a greater number of macrophages and crown –like structures. They were found in CAA-CD, C-HF, and CAA-HF, and rarely found in the C-CD samples. However, they were much more frequent in CAA-HF compared to all other groups, suggesting the greatest level of macrophage infiltration occurs in visceral fat of mice with mutated APP under diet-induced obesity (DIO) conditions.

## Discussion

The cleavage of APP, APP overexpression, and the consequences of APP mutations have been well studied in the brain and muscle for AD pathogenesis and inclusion body myositis [9], [13], [28]–[31], respectively. Researchers have proposed roles for APP in cell adhesion, neuron migration and synaptogenesis [13], [28]. However, it is important to note that APP is not only expressed in the brain and muscle, but in adipose tissue (and other tissues) as well [1], [2], [5]. For several years, there has been a renewed focus on defining the role of APP beyond its association with extracellular plaques and intracellular inclusions, with the hope that understanding the effects of APP in peripheral tissues would potentially provide more information about the link between APP and AD risk. Our results demonstrate novel effects of mutated APP on adipose tissue: severe hypoleptinemia, decreased adiposity, and reduced adipocyte size (Figure 10). These effects were reversed under obesogenic conditions, but this reversal was accompanied by mutant APP-associated increases in insulin resistance, increased circulating triglycerides, and increased macrophage infiltration of visceral adipose tissue (Figure 10).

It has previously been shown that APP expression is upregulated in adipose tissue in response to obesity [1], [2] and APP knock-out mice have reduced body weight [32]. Recent studies have identified that conditions which are associated with reduced adiposity are also associated with reduced amyloid pathology in mouse models expressing APP mutations [33], [34]. Such studies provide correlative support for changes in adiposity modulating specific aspects of amyloid pathogenesis. The current study builds upon these findings by raising the potential for mutations in APP in humans to promote alterations in adipose tissue. These changes in adipose tissue could induce insulin resistance and endocrine alterations which also contribute to adipose-mediated changes in amyloid pathology. Our studies reveal decreased adiposity in a mutant APP mouse model of CAA. However, this novel phenotype was only observed under non-obesogenic conditions, while CAA mice given an obesity-inducing diet exhibited similar adiposity as wild-type mice under the same conditions. Taken together, these data link mutant APP expression to the regulation of adiposity, potentially impairing adipogenesis under non-obesogenic conditions. While the basis for mutant APP to decrease adiposity is not known, it is important to note the significantly reduced adipocyte size in control diet-fed mice. These data suggest that mutant APP is not decreasing adiposity via decreasing the number of adipocytes, but rather primarily through decreased size and potentially decreased differentiation of adipocytes. In order to evaluate changes in adipocyte differentiation due to mutant APP, we evaluated PPAR and C/EBPα mRNA expression in visceral and subcutaneous fat depots. A significant decrease in PPARγ and C/EBPα mRNA was determined due to genotype in subcutaneous fat (Figure 3A +3B) while protein analysis revealed slight increases in these proteins for CAA-CD mice. Coupled with our data on mutant APP altering adipose size, it is likely changes are occurring at the level of adipocyte differentiation. One possibility for altered adipose size in the current study is mutant APP-associated changes in lipase expression. However, it is important to point out that these effects may be adipose depot-specific. Of further note is the lack of adipocyte changes following mutant APP expression *in vitro* (using the 3T3-L1 model), with mutant APP expression induced after differentiation had occurred. Taken together, these data suggest that mutant APP may preferentially mediate its effects in pre-adipocytes, although more work needs to be conducted to experimentally define mutant APP effects on pre-adipocytes.

In addition to the observed changes in adiposity for mice expressing mutant APP, there was also a striking level of hypoleptinemia observed in mutant APP expressing mice. This decrease in leptin was so severe in the present study that it resulted in nearly undetectable levels of leptin in the serum (ELISA analysis). We also detected reduced levels of leptin in subcutaneous and visceral fat depots and 3T3-L1 preadipocytes expressing mutated APP. To our knowledge, these are the first studies to demonstrate such severe hypoleptinemia occurring as a result of mutant APP expression. Furthermore, this level of hypoleptinemia cannot be explained based on the levels of reduced adiposity in CAA mice fed the control diet. Previous studies have reported decreased plasma leptin in the Tg2576 mouse [35], in which APP expression is driven by a hamster prion promoter, resulting in a primary localization of mutant APP in the brain [36]. Additionally, impairments in circulating leptin have been proposed to play a role in the regulation of AD pathogenesis. For example, administration of leptin decreases AD-like pathology in transgenic mouse models of AD pathology (TgCRND8 and Tg2576) [35], [37], with increased AD risk in human patients reported for subjects with low levels of circulating leptin [38]. CAA mice fed the HF diet exhibited a reversal of hypoleptinemia, exhibiting comparable levels of leptin to C-HF mice. The ability of CAA-HF leptin levels to reach C-HF leptin levels provides evidence that the systems responsible for regulating leptin are intact in this mouse model. The basis for leptin downregulation under CD conditions, as well as the apparent selectivity of mutant APP to modulate leptin over other adipokines, are unknown and must be further explored. However, it is well known that leptin expression is dependent on adipocyte size, as shown in rodent and human studies [39]–[41].

While the high level of lipids in adipose tissue made Aβ measurements impossible, recent studies suggest that APP processing in adipose tissue may be important to the regulation of adiposity. For example, a recent study has shown that transgenic mice lacking BACE1 (β-site amyloid precursor protein-cleaving enzyme 1), an essential enzyme for APP processing, are leaner than wild-type controls and have less adipose tissue [42]. Coupled with the findings in APP outlined above, it is clear that BACE1 and APP are both involved in regulating adipogenesis and adipocyte homeostasis. The present study suggests that the ultimate impact of changes in APP or BACE1 expression, or the ultimate impact of mutations in these genes, is dictated by caloric intake and energy expenditure. In conditions which are obesogenic (excess calories, reduced energy expenditure), and that have altered expression of APP or APP processing components, there is likely a promotion of adiposity and exacerbation of obesity-induced metabolic dysfunction as the result of impaired adipose function. Conversely, under non-obesogenic conditions and altered APP expression, these same genes result in impaired endocrine homeostasis due to impaired adipose function. It is well known that BACE and APP expression are highly related to the genesis of Aβ: whereby when BACE is moderately overexpressed, APP is processed to generate more Aβ [43] and when BACE is genetically ablated, Aβ is not produced [44]. Interestingly, the leptin promoter contains numerous regulatory elements [45], [46] that are also involved in the expression and processing of APP, suggesting complex interactions between multiple signaling pathways and the effects of mutant APP on adipose tissue function. For example, Sp1 regulates BACE1 expression [47] and HIF-1 regulates APP and BACE1 expression [48]–[50]. These regulatory elements are also known to be modulated in response to diet [27], [51], [52], providing further evidence for a connection between APP expression and adiposity.

While CAA-HF mice were able to reverse certain phenotypes found in the CAA-CD mice, they also presented evidence for an exacerbation of obesity-induced metabolic dysfunction including insulin resistance, elevated triglycerides, and macrophage infiltration of adipose tissue. The factors leading to insulin resistance are multifactorial, but numerous studies have linked the development of insulin resistance to increased levels of inflammation [53], [54]. Obesity is often accompanied by insulin resistance and inflammatory visceral fat tissue [17], [19], [55]. However, it is intriguing that the CAA-HF mice revealed this phenotype whereas the C-HF mice did not, suggesting an increased susceptibility for the CAA genotype. With the epidemic proportion of obese individuals in the United States, and soon to be worldwide [56], we hypothesize this phenotype to be more common among individuals with genetic mutations of APP. This supports the current findings that Alzheimer’s disease is correlated with obesity and type II diabetes [57]–[60].

In conclusion, novel metabolic abnormalities have been demonstrated in this study for the CAA mouse under two different dietary conditions. This data has implications for the prevalence and risk of metabolic disturbances in human subjects and presents new consequences for APP mutations beyond Alzheimer’s disease risk. Numerous rodent studies have also demonstrated increased amyloid plaque deposition, cognitive impairment, and increased APP expression due to consumption of a high fat diet or under the conditions of diet-induced obesity (DIO) [2], [61], [62]. Altogether, this data provides further evidence for a link between adiposity and APP biology.
